# The effect of phosphoric acid etching duration on the bond strength of universal adhesive on enamel with or without erosion

**DOI:** 10.3389/fdmed.2025.1685381

**Published:** 2025-10-20

**Authors:** Shasha Huang, Yuan Chai, Guangliang Niu

**Affiliations:** ^1^ Department of Oral and Maxillofacial Surgery, the First Affiliated Hospital of Nanchang University, Nanchang, China; ^2^ Department of Stomatology, Beijing Integrated Traditional Chinese and Western Medicine Hospital, Beijing, China; ^3^ Department of Dental Materials Lab, Peking University School and Hospital of Stomatology, Beijing, China; ^4^ Dental Medical Devices Testing Center of Peking University School of Stomatology, Beijing, China

**Keywords:** dental erosion, enamel, shear bond strength, universal adhesives, phosphoric acid

## Abstract

This study evaluated the effect of phosphoric acid etching duration on the bond strength of a universal adhesive (UA) on enamel, both with and without erosion (0.3% citric acid with a pH of 2.6 for 5 min four times a day for five consecutive days). Flat, polished bovine incisors (*n* = 60) were divided into two groups according to different enamel conditions, and were further divided into three subgroups (*n* = 10) according to phosphoric acid etching duration: 0 [self-etch (SE) mode], 3, and 15 s [etch-and-rinse (ER) mode]. A shear bond strength (SBS) test was performed after applying the UA and composite blocks. Surface topography and roughness were assessed after the phosphoric acid treatment of the incisors, with or without erosion, using scanning electron microscopy and a profilometer, respectively. In the normal enamel group, the SBS values were significantly higher in the ER mode group, while neither the phosphoric acid etching duration nor the etching mode affected the SBS or roughness in the eroded enamel group. In summary, applying UA after phosphoric acid etching using the SE mode may be sufficient for eroded enamel, while reducing the phosphoric acid etching duration to 3 s may be acceptable in resin restoration with normal enamel.

## 1 Introduction

In recent decades, the increasing prevalence of dental erosion (30.4%) has received more attention from clinicians and researchers (1). Dental erosion is defined as the gradual chemical loss of dental hard tissue that does not involve any microorganisms (2). Apart from reducing the mineral content of enamel, erosive challenges also decrease its hardness (3), highlighting the need for appropriate preventive procedures and restorative treatments.

The development of adhesive dentistry has advanced significantly since the introduction of the concept of acid etching by Buonocore in 1955. Dental adhesives have evolved from total etch systems (fourth and fifth generations) to self-etch (SE) (sixth and seventh generations) systems, with the former requiring more etching using phosphoric acid gel (4). Recently, a novel family of adhesives, known as universal (UAs) or multi-mode adhesives, has become widely used in dental clinics due to their multifunctionality and simplicity. They can be applied with or without an extra etching step (5) and on different substrates (6, 7). Bonding to enamel has been considered a reliable and durable method for composite restoration since the introduction of the acid etching technique. Etching enamel for 15 s with phosphoric acid before applying UA is generally recommended (8, 9). Interestingly, recent studies have revealed that shortening the phosphoric acid etching duration to 3 s did not adversely affect the enamel bond strength or durability of the UA when using the etch-and-rinse (ER) mode; however, pre-etching for 15 s is still widely used in clinical practice (10, 11).

Based on this, it is unknown whether such a reduced phosphoric acid etching duration is also applicable to eroded enamel. There are few studies available on the restoration of eroded enamel using UA with different etching modes (12), especially those that focus on the combined factors of UA etching modes and phosphoric acid etching duration. Therefore, the effect of phosphoric acid etching duration before applying UA to normal and eroded enamel was investigated in this study. The null hypotheses tested were as follows: (1) the shear bond strength (SBS) of UA when using the ER mode will be higher than when using the SE mode, regardless of enamel condition (normal or eroded), and (2) reducing the phosphoric acid etching duration from 15 to 3 s will not impair the SBS of UA when using the ER mode, regardless of enamel condition (normal or eroded).

## 2 Materials and methods

### 2.1 Specimen preparation and groups

In total, 60 fresh, extracted bovine incisors were stored in a 0.1% thymol solution at 4°C with a pH of 7.0. The incisor roots were removed using a low-speed diamond blade (XL 12205, Benetec Ltd., London, UK) coupled to a straight handpiece. The crowns were embedded in self-curing acrylic resin (Jet Clássico, São Paulo, SP, Brazil) in polyvinyl chloride ring molds. The exposed labial surfaces were ground flat for 1 min using 320-grit silicon carbide sandpaper (Extec Corp., Enfield, CT, USA) mounted on a circular polishing machine (LaboPol-30, Struers ApS, Ballerup, Denmark) with constant water cooling until an area of enamel approximately 7 mm^2^ in size was exposed. All the polished teeth were washed and air-dried at room temperature for 24 h.

### 2.2 Erosive challenge

After grinding, the specimens (*n* = 60) were randomized and allocated into two groups (*n* = 30 each) according to the following enamel condition: normal (without erosion) or eroded (after erosive challenge). The normal specimens were placed in artificial saliva that was changed daily for 5 days. The eroded group was treated with 0.3% citric acid (pH 2.6) for 5 min four times a day for five consecutive days (13). The citric acid solution was prepared by dissolving anhydrous citric acid powder (Sigma-Aldrich, Poole, Dorset, UK) in deionized water. Between each immersion, the specimens were rinsed with deionized water for 20 s and placed in artificial saliva for 1 h. After the final immersion, the specimens were kept in artificial saliva overnight. The artificial saliva composition was based on a study from the literature (14).

### 2.3 Restorative procedure

Each enamel condition group was further randomized and divided into three subgroups (*n* = 10) according to the 35% phosphoric acid (Ultradent Products, South Jordan, UT, USA) application duration (0, 3, or 15 s). The non-pre-etched group (0 s) was regarded as the SE group, and the pre-etched groups (3 or 15 s) were regarded as the ER groups. After etching, the surface was rinsed with deionized water for 15 s and air-dried. A piece of double-sided adhesive tape with a 3 mm diameter hole was used to define the enamel’s adhesive area. The adhesive system (Scotchbond UA, 3M Oral Care, St. Paul, MN, USA) was actively applied for 20 s, air-dried, and light-cured for 10 s using an LED device (Radii-Cal, SDI, Bayswater, VIC, Australia) with a power output of 600 mW/cm^2^. Composite (Filtek Universal Restorative, 3M Oral Care, St. Paul, MN, USA) was built up on the specimen’s surface using Teflon molds with an inner diameter of 3 mm and a height of 2 mm to define the restoration areas. Additional light curing of the resin block was performed after removing the mold. All the procedures were carried out by a single trained operator. The completed specimens were stored in distilled water at 37°C for 24 h. Table 1 presents the specifications of the materials used.

**Table 1:** Materials used in the restorative procedures.

Name	Material	Manufacturer	Composition	Batch number
Scotchbond UA	UA system	3M ESPE (St. Paul, MN, USA)	MDP monomer, dimethacrylate resins, HEMA, methacrylate-modified polyalkenoic acid copolymer, filler, ethanol, water, initiators, silane	6806584
Etch 35 Gel	Phosphoric acid gel 35%	Kulzer GmbH (Hanau, Hesse, Germany)	Phosphoric acid gel 35%	K010182
Filtek Universal Restorative	Nanohybrid composite	3M ESPE (St. Paul, MN, USA)	Resin matrix: Bis-GMA and diluents Filler (silicone dioxide)	NA62338

UA, universal adhesive; MDP, methacryloyloxydecyl dihydrogen phosphate; HEMA, hydroxyethyl methacrylate; Bis-GMA, bisphenol A glycidyl methacrylate.

### 2.4 SBS test

The SBS of the specimens after the restoration was tested. The specimens were loaded to failure at a crosshead speed of 1.0 mm/min using a universal testing machine (Instron 3367, Norwood, MA, USA). A metal rod with a chisel-shaped end was used to apply the load to the resin composite cylinder directly adjacent to the flat surface of the ground tooth. The SBS value (MPa) was calculated using the peak load at failure divided by the bonded area. After testing, the specimens were observed under an optical microscope (MZ16, Leica Microsystems, Heerbrugg, St. Gallen, Switzerland) at a magnification of 20× to examine the bond failure site. The fracture layer of the resin surface with adherent enamel and visible residue was estimated to classify the failure mode as adhesive, enamel cohesive, resin composite cohesive, or mixed (partially adhesive and partially cohesive).

### 2.5 Roughness in different enamel conditions after various pre-etching durations

To assess surface roughness in the two enamel conditions with different phosphoric acid etching durations, 60 specimens (*n* = 10) were prepared as previously described, but without applying the adhesive or resin composite. The average roughness (Ra) was measured by a blind operator using a profilometer (MarSurf PS1, Mahr, Esslingen, Baden-Württemberg, Germany), with the stylus tip moving perpendicularly on the surface. An average of three readings was taken for all the specimens.

### 2.6 Scanning electron microscopy

Scanning electron microscopy (SEM) (JSM-1900F, JEOL Ltd., Akishima, Tokyo, Japan) was conducted to assess the morphological changes due to the different enamel conditions and phosphoric acid etching durations. Six enamel specimens were prepared without applying the adhesive and resin composite, consisting of two enamel conditions (ground and eroded) and three phosphoric acid etching durations (0, 3, or 15 s). The specimens were dehydrated using a series of immersions in increasing concentrations of aqueous tert-butanol (50% for 20 min, 75% for 20 min, 95% for 20 min, and 100% for 2 h) and were then placed directly into a critical point dryer (ID-3, Elionix, Tokyo, Japan) for 30 min. Finally, the specimens were coated with a thin film of gold in a vacuum evaporator (Quick Coater Type SC-701, Sanyu Electron, Tokyo, Japan). The SEM observations were carried out using an operating voltage of 10 kV.

### 2.7 Statistical analysis

For sample size calculation, a pilot trial was conducted on normal and eroded enamel with five specimens in each subgroup. A one-way ANOVA was used to calculate the total sample size required for each enamel condition using G*Power software (version 3.1), with an alpha level of 0.05 and a power of 0.80. Based on the above parameters, nine and three specimens were needed for the eroded and normal enamel groups, respectively. For consistency, 10 specimens were selected for each subgroup.

The normality and assumption of equal variance of the data were checked before the SBS and roughness analyses were conducted using a two-way ANOVA to evaluate the effects of enamel condition and phosphoric acid etching duration on the SBS of enamel. *Post hoc* pairwise comparisons were performed using the Bonferroni test. All the analyses were performed using SPSS Statistics software version 22.0 (IBM, Armonk, NY, USA) with a significance level of *p* < 0.05.

## 3 Results

### 3.1 Shear bond strength

**Figure 1:**
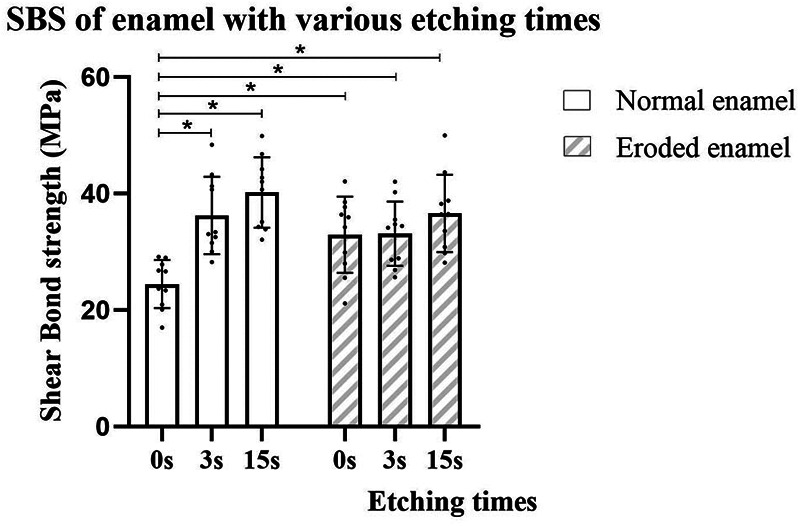
Shear bond strength (MPa) of UA on enamel with or without erosion with different phosphoric acid pre-etching durations.

The SBS testing results for the composite when bonded to ground and eroded enamel, respectively, are presented in Figure 1 and Table 2. The two-way ANOVA revealed that pre-etching duration significantly influenced the SBS values (*p* < 0.001); however, there was no difference between the enamel conditions (*p* > 0.05). The interaction between the two factors was significant (*p* < 0.05). For normal enamel, the SBS was higher in the ER mode group compared to the SE mode group (*p* < 0.05), and the SBS of UA in pre-etched groups was not influenced by the pre-etching duration (3 or 15 s). For the eroded enamel, no difference was found between the groups with different UA etching modes or phosphoric acid etching durations. Furthermore, the SBS of the eroded enamel in the SE mode group was significantly higher than that of the normal enamel in the SE mode group (*p* < 0.05). However, no difference in SBS in the different pre-etched groups was observed, regardless of the enamel condition.

**Table 2:** Shear bond strength (MPa) of UA on enamel with or without erosion after different phosphoric acid pre-etching durations.

Pre-etching duration (s)	Normal enamel (MPa)	Eroded enamel (MPa)
0	24.44 ± 4.13^a^ (21.48–27.39)	32.93 ± 6.56^b^ (28.24–37.62)
3	36.21 ± 6.65^b^ (31.45–40.97)	33.10 ± 5.53^b^ (29.15–37.06)
15	40.16 ± 6.04^b^ (35.84–44.48)	36.58 ± 6.63^b^ (31.84–41.33)

The means and standard deviations of the shear bond strength, with the 95% confidence intervals in brackets, are presented. The same lowercase letter in superscript indicates no significant difference (*p* > 0.05).

### 3.2 Failure mode analysis

The failure mode results after SBS testing are shown in Table 3. A predominance of adhesive failures for all the SE groups was observed for both enamel conditions, while the ER groups had more mixed failures regardless of etching duration (3 or 15 s). There were some resin cohesive failures (10%) that occurred in the eroded enamel etched groups (3 or 15 s).

**Table 3:** Failure mode analysis of the specimens after shear bond strength testing.

Failure mode	Normal enamel			Eroded enamel		
	0 s	3 s	15 s	0 s	3 s	15 s
Adhesive failure/dentin cohesive failure /composite cohesive failure/mixed failure (percentages)	[100/0/0/0]	[60/0/0/40]	[60/0/0/40]	[100/0/0/0]	[50/0/10/40]	[50/0/10/40]

### 3.3 Roughness in different enamel conditions after various pre-etching durations

**Figure 2:**
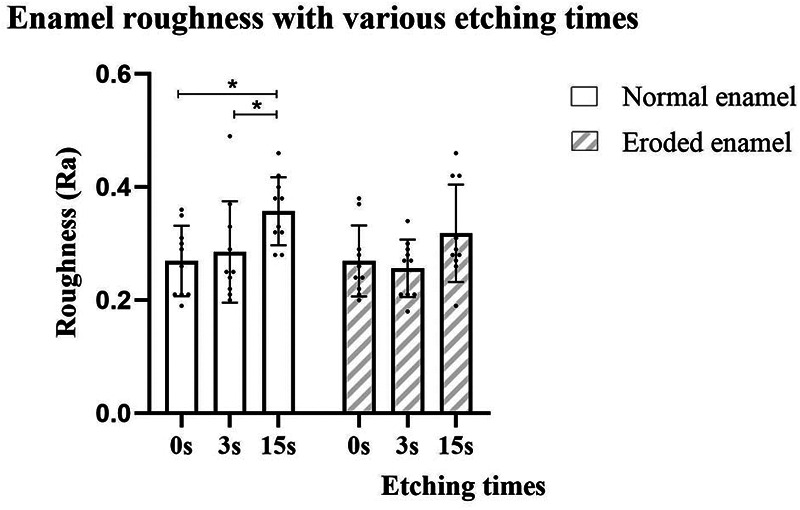
Roughness (µm) of enamel surfaces with or without erosion and with different phosphoric acid pre-etching durations.

Comparisons of surface roughness among the groups are shown in Figure 2 and Table 4. The two-way ANOVA revealed that the phosphoric acid etching duration significantly influenced the roughness of the enamel (*p* < 0.01); however, there were no differences among enamel conditions or their interactions (*p* > 0.05). Phosphoric acid etching duration (0, 3, or 15 s) had no impact on the roughness of the eroded enamel (*p* > 0.05), while etching for 15 s significantly increased the roughness of the normal enamel compared to that in the SE mode and etching for the 3 s group (*p* < 0.05). Regarding enamel conditions, only the non-etched group was statistically different, with the eroded enamel group having higher roughness values than the normal enamel group (*p* < 0.05). No difference in roughness was found among the etched groups (3 or 15 s), regardless of enamel condition (*p* > 0.05).

**Table 4:** Effect of pre-etching duration on the Ra of different enamel conditions (normal or eroded) in µm.

Pre-etching duration (s)	Normal enamel (µm)	Eroded enamel (µm)
0	0.269 ± 0.062A^a^ (0.225–0.313)	0.269 ± 0.063A^a^ (0.224–0.314)
3	0.285 ± 0.090A^a^ (0.221–0.349)	0.256 ± 0.051A^a^ (0.220–0.292)
15	0.357 ± 0.060A^b^ (0.314–0.400)	0.318 ± 0.086A^a^ (0.256–0.380)

The roughness means and standard deviations, with 95% confidence intervals in brackets, are presented. The same lowercase letter in superscript within the same column indicates no significant difference (*p* > 0.05). The same uppercase letter in superscript within each row indicates no significant difference (*p* > 0.05).

### 3.4 SEM observations

**Figure 3:**
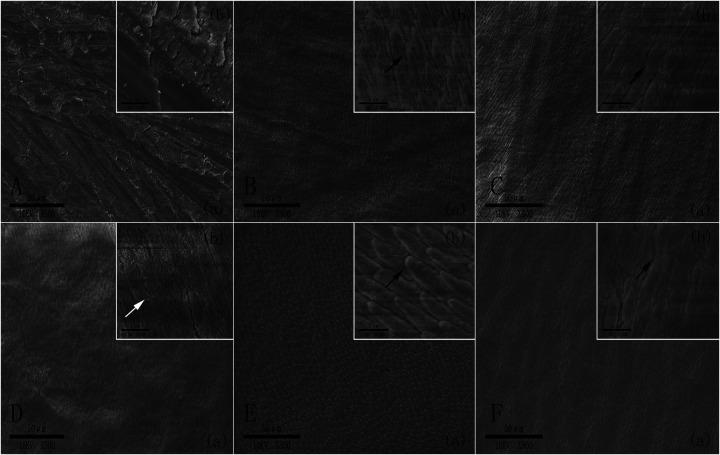
Representative SEM images of normal/eroded enamel after the phosphoric acid treatment with different etching durations. (**A**) Normal enamel without etching. (**B**) Normal enamel treated with phosphoric acid for 3 s. (**C**) Normal enamel treated with phosphoric acid for 15 s. (**D**) Eroded enamel treated without etching. (**E**) Eroded enamel treated with phosphoric acid for 3 s. (**F**) Eroded enamel treated with phosphoric acid for 15 s. (a) low magnification (×500) and (b) high magnification (×1,000). The white arrow indicates the spicular etching pattern of the enamel rods, and the black arrows indicate the characteristic etching pattern of the enamel rods.

Representative SEM images of enamel specimens with different enamel conditions (normal or eroded) and phosphoric acid etching durations (0, 3, or 15 s), without applying UA and resin composite, are shown in Figures 3A–F. In the ground enamel without etching (Figure 3A), periodic grooves and debris from the carbide polishing papers were observed, while a typical enamel etching pattern was found in the enamel with phosphoric acid pre-etching, regardless of its condition (Figures 3B,C,E,F). Images of eroded enamel without pre-etching exhibited a pronounced etching pattern with spicular shapes and clearer enamel rod edges compared to the non-etched ground enamel group (Figure 3D).

## 4 Discussion

In this study, phosphoric acid etching duration and different enamel surface conditions were taken into consideration collectively to explore which strategies are better for normal and eroded enamel when using UA in resin restoration.

In the normal enamel group, the SBS of the UA ER mode groups performed better, especially for the shortest etching duration (3 s), which had similar efficacy to the traditional etching duration (15 s). The characteristic etching pattern of the pre-etched enamel found in the SEM may explain the improvement in SBS. It is widely acknowledged that the “tag-like” penetration of resin into etched enamel is the major mechanical retention force between bonding materials and enamel. Furthermore, the phosphoric acid treatment improves the wettability and bonding area of the adherent surface (15), leading to deeper and wider penetration of the resin. In addition, the increased SBS values in the ER mode groups are supported by an increased number of mixed failures compared to adhesive failures alone in the SE mode group. However, increasing the etching duration to 15 s did not improve SBS further, which may be due to less prominent discrepancies in the honeycomb enamel etching pattern. Similarly, there are three studies on the SBS of UA on human enamel (10, 11, 16) and two studies on that of UA on bovine teeth (17, 18), covering five phosphoric acid etching durations (0, 1, 5, 10, and 15 s). All of these studies showed that using the ER mode led to better performance compared to the SE mode, regardless of the UA brand, and no difference was observed between the different durations when using the ER mode. Moreover, one of the studies compared UA with a traditional self-etch adhesive and found that two-step self-etch adhesives were the best when using the SE mode, but there was no difference between the 3 and 15 s etching durations among the adhesives when using the ER mode (11). This was in line with another study (19).

However, there is some inconsistency in the reporting of the performance of different UA etching modes in clinical resin restorations. Some studies reported better performance using the ER mode compared to the SE mode for non-carious cervical lesions (NCCLs) at 1-year (20) and 5-year follow-ups (21). Other studies showed similar performance between the ER and SE modes for NCCLs at 18, 24, and 36 months (22) and at 2-year follow-ups (23). Moreover, one systematic review compared UAs with other traditional adhesives and included 15 studies that covered 12 UAs, 4 self-etch adhesives, and 5 etch-and-rinse adhesives. The review found that UAs achieved comparable performance to conventional adhesives regardless of application mode over follow-up periods ranging from 6 to 48 months (24). Thus, UAs are promising for clinical application compared to traditional self-etch and etch-and-rinse adhesives, although long-term follow-up is still needed.

For the eroded enamel, neither the UA etching mode nor the phosphoric acid etching duration influenced the SBS of the UA. Accordingly, the first null hypothesis was partially rejected, and the second null hypothesis was accepted. The increased SBS obtained in the eroded enamel SE group compared to the normal enamel group may be due to the special erosion morphology verified by SEM. This result was in agreement with other studies (12, 13) that explored the effect of erosion on the bond strength of enamel, with both studies indicating better results for UAs after erosion. Furthermore, the SBS of the eroded enamel had the same trend as the ground enamel with a shorter etching duration, which could be ascribed to the similar etching patterns observed. Interestingly, the eroded enamel group had slightly fewer adhesive failures (10%) compared to the normal enamel ER mode groups, implying increased bonding in the eroded enamel group. Although the SBS in the eroded enamel SE mode group was higher than that in the normal enamel SE mode group, all samples had adhesive failures, which indicated relatively weaker bonding compared to the ER mode groups.

Although some studies have investigated the effect of different phosphoric acid pre-etching durations on resin restoration of eroded enamel, few have compared the UA SE and ER application modes. Karadas found greater microshear bond strength (μSBS) in UAs when applied to demineralized enamel using the ER mode compared to the SE mode (25). This result was different from that of our study, which may be due to the different erosion model used in our study. A systematic review compared the bond strength of UAs, traditional self-etch adhesives, and etch-and-rinse adhesives to eroded enamel and found that the etching modes of the adhesives had no impact on their bond strength (26). This is in line with our study, suggesting it is possible to use the SE mode to apply adhesive to eroded enamel.

In terms of surface roughness, the values increased with etching duration (15 s) in the ground enamel group but remained stable in the eroded enamel group. This may explain the stable SBS across different etching durations in the eroded enamel group, as well as the similar morphology revealed by SEM. The roughness of the normal enamel was consistent with the findings of Shimatani et al. (17) and Salman and Hussein (15). However, roughness alone cannot fully explain the increased SBS in the normal enamel group when etching for 3 s, as similar roughness values were found in the etching for 3 s and the non-etched groups. These results suggest that other factors, such as better surface free energy and polarity triggered by acid etching, can synergistically improve the bond strength of normal enamel (15). Moreover, morphological characteristics and geometric surface area may have a stronger impact on SBS than roughness, as the obvious honeycomb etching pattern of the normal enamel after etching was not found in the SE mode group. This finding was in line with that of Tsujimoto et al. (16).

Moreover, the sufficient and reliable chemical bonding provided by the functional monomers in UAs may reduce the need for longer phosphoric acid etching duration. Apart from the mechanical bonding, chemical bonding plays an important role in the bonding efficacy of UAs. It has been reported that methacryloyloxydecyl dihydrogen phosphate (MDP), a key acidic functional monomer in the majority of UAs, forms a stable nanolayer and deposits stable MDP-calcium salts at the adhesive interface that helps to improve bond quality (27). Moreover, phosphoric acid etching promotes greater chemisorption of the MDP monomer by enamel crystals compared to a non-etched surface (28). Furthermore, the increased surface free energy and polarized enamel induced by the phosphoric acid mentioned above also create a more reactive surface for chemical bonding (29). In addition to the MDP monomer, the polyalkenoic acid copolymer (Vitrebond copolymer, 3M ESPE) contained in Scotchbond UA is able to chemically bond to hydroxyapatite (30), which may also play a role in the stable SBS values when using the lowest etching duration.

Based on the results of this study, useful suggestions can be provided for dental clinical practice. When restoring ground enamel with a UA, phosphoric acid etching for 3 s is more efficient; for patients with dental erosion, restoration using the SE mode and avoiding etching is optimal. This bonding strategy has higher efficacy as it helps to reduce time spent chair-side, decrease the chance of surface contamination, minimize subsurface dissolution of enamel, and decrease the risk of over-etching and postoperative sensitivity in patients, which is a common concern in clinical practice. In this case, selective enamel etching may not always be the optimal option for bonding UAs to eroded enamel.

Nevertheless, this study had some limitations that require further investigation. First, this was an *in vitro* study that could not fully mimic the *in vivo* environment. For example, we used a simplified erosion model, which is different from oral cavities with pH fluctuations and saliva. Citric acid was selected because it is one of the most commonly consumed food acids in acidic diets, which are recognized as one of the major contributors to extrinsic dental erosion. Second, only the short-term performance of applying UAs to enamel was assessed; thus, further exploration of their long-term behavior using artificial aging, such as thermocycling (6) and mechanical load (31), is required. However, aging with underlying clinical supporting data is of vital importance, as the majority of aging methods lack clinical validation (32). Finally, only a small number of bovine teeth were used, as they most closely resemble human teeth in chemical composition (33). Bovine teeth showed the same results in several studies on the effect of different phosphoric acid etching times on SBS when compared to human teeth, as mentioned previously, although teeth of bovine origin have a relatively lower Ca/P ratio than human teeth.

## 5 Conclusion

This study concludes that pre-etching normal enamel with phosphoric acid for 3 and 15 s results in similar SBS in resin restoration, and that the SBS is significantly higher than that of the UA self-etch mode. In the eroded enamel group, neither the phosphoric acid etching duration nor the UA etching mode impacted the SBS, which could be explained by the similar etching patterns and roughness obtained. These findings suggest that using the UA SE application mode may be sufficient for eroded enamel and that reducing the phosphoric acid etching duration to 3 s may be acceptable for resin restorations of normal enamel. Consequently, the bonding strategy may need to be adjusted when encountering different enamel conditions to obtain optimal efficiency. Future research should explore the long-term bonding performance of these bonding strategies to confirm their durability and reliability.

## Data Availability

The raw data supporting the conclusions of this article will be made available by the authors, without undue reservation.
